# Genome-wide characterization and expression analysis of geranylgeranyl diphosphate synthase genes in cotton *(Gossypium spp.)* in plant development and abiotic stresses

**DOI:** 10.1186/s12864-020-06970-8

**Published:** 2020-08-15

**Authors:** Faiza Ali, Ghulam Qanmber, Zhenzhen Wei, Daoqian Yu, Yong hui Li, Lei Gan, Fuguang Li, Zhi Wang

**Affiliations:** 1 State Key Laboratory of Cotton Biology, Key Laboratory of Biological and Genetic Breeding of Cotton, Institute of Cotton Research, Chinese Academy of Agricultural Sciences, Anyang, 455000 China; 2 Zhengzhou Research Base, State Key Laboratory of Cotton Biology, Zhengzhou University, Zhengzhou, 450001 China

**Keywords:** GGPP, *Gossypium hirsutum*, Gibberelline, *cis*-elements, Sequence logos, Collinearity

## Abstract

**Background:**

GGPP (geranylgeranyl diphosphate) is produced in the isoprenoid pathway and mediates the function of various plant metabolites, which is synthesized by GGPPS (GGPP synthases) in plants. *GGPPS* characterization has not been performed in any plant species except *Arabidopsis thaliana*. Here, we performed a complete computational and bioinformatics analysis of *GGPPS* and detected their transcription expression pattern in *Gossypium hirsutum* for the first time so that to explore their evolutionary relationship and potential functions. Finally, we unravelled evolutionary relationship, conserved sequence logos, gene duplication and potential involvement in plant development and abiotic stresses tolerance of *GGPPS* genes in *G. hirsutum* and other plant species.

**Results:**

A total of 159 *GGPPS* genes from 18 plant species were identified and evolutionary analysis divided these *GGPPS* genes into five groups to indicate their divergence from a common ancestor. Further, *GGPPS* family genes were conserved during evolution and underwent segmental duplication. The identified 25 *GhGGPPS* genes showed diverse expression pattern particularly in ovule and fiber development indicating their vital and divers roles in the fiber development. Additionally, *GhGGPPS* genes exhibited wide range of responses when subjected to abiotic (heat, cold, NaCl and PEG) stresses and hormonal (BL, GA, IAA, SA and MeJA) treatments, indicating their potential roles in various biotic and abiotic stresses tolerance.

**Conclusions:**

The *GGPPS* genes are evolutionary conserved and might be involve in different developmental stages and stress response. Some potential key genes (e.g. *GhGGPP4, GhGGPP9,* and *GhGGPP15*) were suggested for further study and provided valuable source for cotton breeding to improve fiber quality and resistant to various stresses.

## Background

Isoprenoids represent the largest group of biologically active and specialized metabolites in plants. Many Isoprenoids provides protection to plants from pathogens and herbivores threats [1]. Isoprenoids also have important functions in plant photosynthesis and respiration processes and involve many hormonal pathways (abscisic acid, brassinosteroids, cytokinin, gibberellin, and strigolactones) important for development and growth regulation in plants [1–4].

In isoprenoid pathway, prenyl diphosphate synthases are active with important isozymes for isoprenoid metabolism, use isopentenyl diphosphate (IPP) and dimethylallyl diphosphate (DMAPP) as substrates in the mevalonate (MVA) or methylerythritol (MEP) pathway. In general, they are represented by three enzymes such as geranyl diphosphate synthase (GPPS), farnesyldiphosphate synthase (FPPS) and geranylgeranyl diphosphate synthase (GGPPS) in plants [4].

*GGPPS* is an essential enzyme among three isoprenoids for primary and secondary isoprenoid compounds synthesis such as chlorophylls, carotenoids and derivatives including different hormones (e.g. abscisic acid (ABA), strigolactones and gibberellins), and proteins (e.g. plastoquinones, ubiquinones, phylloquinones, tocopherols, diterpenoids, polyprenols, dolichols, and prenylated) [4]. *GGPPS* exerts successive additions of IPP to DMAPP, GPP and FPP as a homodimer [5] and have been studied in many organisms such as bacteria [6], yeast [7], fungi [8], plants [9], mammals [10] and insects [11].

In *A. thaliana,* 12 *GGPPS* genes were identified [12] and have been reported with their basic characterization date back more than a decade ago [4, 9, 13–15]. It is reported previously that 10 *GGPPS* paralogs (*GGPPS1- GGPPS4* and *GGPPS6-GGPPS11*) out of 12 showed GGPPS activity and are functional in *A. thaliana*, however, *GGPPS12* gene was studied in two works which demonstrated that *GGPPS12* lack GGPPS activity [9, 15].

Furthermore, the *GGPPSs* were found in different cell organelles in *A. thaliana* Such as *GGPPS1* existed in mitochondria, *GGPPS4* in the ER (endoplasmic reticulum), and *GGPPS2* and *GGPPS6-GGPPS11* in plastids [9, 13–15]. In addition, *GGPPS* genes showed differential spatio-temporal expression both by Northern analysis and GUS expression of *GGPPS* promoters such as photosynthetically active tissues were found to be rich with *GGPPS11* [4, 9] and provided GGPP as a substrate for biosynthesis of essential photosynthesis related isoprenoid compound. Similarly, *GGPPS1*- *GGPPS10* were specifically expressed in root or seed tissues and are important during plant development [4]. Additionally, on the base of sequence analysis, *GGPPS5* was proposed to be a pseudo gene [4], whereas *GGPPS12* was almost different paralog from all predicted 12 *GGPPSs* in *A. thaliana*, and does not exhibit GGPP synthase activity [4, 9, 15]. However, *GGPPS12* together with *GGPPS11* was active as a heterodimer and can synthesize GPP [15].

Dicotyledonous plants diverged from their ancestors about 10–15 million years ago (MYA). The genus *Gossypium* contained 50 species and one of them is *Gossypium hirsutum*. *G. hirsutum* is an allotetraploid plant known as upland cotton. Researchers demonstrated that *G. hirsutum* emerged about 1–2 MYA as a result of an intergenomic hybridization event between *G. arboreum* and *G. raimondii* (A and D genomes) [16–18]. Cotton (*Gossypium hirsutum L.*) is the greatest source of natural fiber and is cultivated worldwide [19] as an important raw material for textile industry. There are many environmental and hormonal stresses that affect cotton growth and productivity which result in low fiber quality and yield. Advancements in cotton genome sequencing [20, 21] make it possible to conduct a complete and comprehensive investigation of cotton genes involve in plant growth and development.

Due to the important role of *GGPPS* for isoprenoid biosynthesis, we investigated the evolutionary relationships of the *GGPPS* genes in 17 plant species, representing major plant lineages (green algae, mosses, gymnosperms, and angiosperms) using a combination of computational analyses. Particularly, we performed a comprehensive analysis of entire *G. hirsutum GGPPS* family members and identified 25 *GGPPS* genes. In our study, we performed a systematic analysis of *GhGGPPS* genes using phylogenetic analysis. The biophysical properties, sequence logos, exon/intron and protein motif distribution, promoter *cis*-element analysis, chromosomal distribution, gene duplication, and colinearity analysis were performed. Moreover, tissue-specific expression patterns as well as abiotic and hormonal stress responses were also investigated for *G. hirsutum GGPPS* genes. The present study provides a foundation and deepens our understanding about molecular and biological functions of *GGPPS* genes in cotton.

## Results

### Identification of *GGPPS* genes in plants

A total of 159 *GGPPS* genes were identified from 18 plant species including green algae, mosses, fern, gymnosperms and angiosperms (Additional file 1:Table S1). Among these, one *GGPPS* gene was identified from *C. reinhardtii,* two from *S. bicolor* and *P. patens,* three from *S. moellendorfii* and *M. truncatula,* four from *Z. mays* and *V. vinifera,* five from *T. cacao,* six from *S. tuberosum,* seven from *G. max* and *P. taeda,* eight from *O. sativa,* 10 from *G. arboreum,* and *G. raimondii,* 12 from *Arabidopsis,* 25 from *B. napus, G. barbadense* and *G. hirsutum.* It is observed that from 18 species, *GGPPS* genes range from 1 *(C. reinhardtii)* to 25 *(G. hirsutum* and *B. napus)* indicating that *GGPPS* gene family underwent expansion during the evolution in land plants. Further, *G. barbadense* and *G. hirsutum* contained maximum (25) number of *GGPPS* genes among four cotton species, indicating the polyploidization and duplication effect on *GhGGPPS* gene family in *G. barbadense* and *G. hirsutum*.

### Evolutionary analysis of *GGPPS* genes

**Fig. 1:**
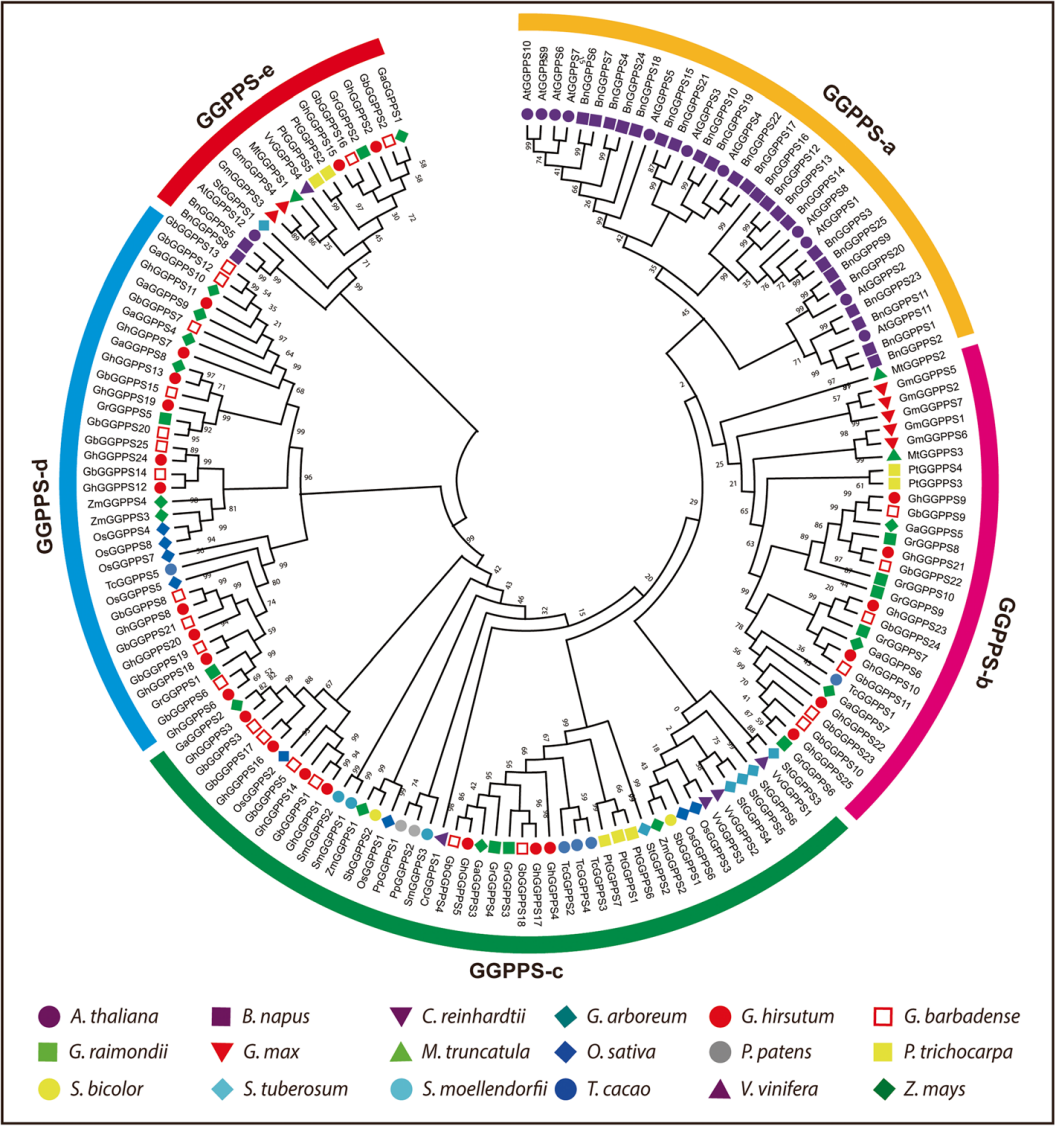
Evolutionary relationship among 159 *GGPPS* genes from 18 plant species. Prefixes At, Os, Sb, Gm, Sm, Pp, Cr, Zm, Pt, Vv, St, Mt, Bn, Tc, Ga, Gr, Gb, and Gh were used to recognize the GGPPS genes from *Arabidopsis, O. sativa, S. bicolor*, *G. max, S. moellendorfii, P. patens, C. reinhardtii, Z. mays*, *P. taeda, V. vinifera, S. tuberosum, M. truncatula, B. napus, T. cacao, G. arboretum, G. raimondii, G. barbadense,* and *G. hirsutum* respectively

Next, we investigated the evolutionary relationships among 159 *GGPPS* genes of all observed plant species to assume evolutionary mechanisms leading to the formation and maintenance of multiple gene copies within them. The evolutionary analysis divided the *GGPPS* genes into five main groups, referred to as GGPPS-a to GGPPS-e (Fig. 1). Group GGPPS-c with maximum number (45 genes) of *GGPPS* family genes from fourteen species while group GGPPS-e with minimum number (16 genes) of *GGPPS* genes from 11 species except *S. bicolor*, *S. moellendorfii, P. patens, C. reinhardtii, Z. mays*, *O. sativa,* and *T. cacao*. Moreover, group GGPPS-b contained 30 genes from eight species including *G. max, P. taeda, G. barbadense, M. truncatula, T. cacao, G. arboreum, G. raimondii,* and *G. hirsutum* while, group GGPPS-a contained 34 genes from 2 species including *Arabidopsis,* and *B. napus* demonstrating a close relationship of these two species*.* Similarly, Group GGPPS-d contained 34 genes from seven species including *O. sativa, Z. mays*, *T. cacao, G. barbadense, G. arboreum, G. raimondii,* and *G. hirsutum*. Interestingly, 159 *GGPPS* genes from 18 species were randomly distributed to all groups, and most orthologous and paralogous genes between allotetraploids and diploids were clustered close to each other in the same group, showing the expansion and evolutionary relationship of the *GGPPS* gene family. The phylogenetic analysis showed that group GGPPS-c contained one *GGPPS* gene from first land plant green algae indicating that *GGPPS* gene family is derived from common ancestor, which is in agreement with earlier studies that all trans-isoprenyl diphosphate synthases are derived from a common ancestor [22, 23]. Similarly, group GGPPS-c also contained two genes from moss and three genes from fern, the more number of *GGPPS* genes from moss and fern than algae indicating that the *GGPPS* genes expanded in plant species during evolution by duplication event. Group GGPPS-a, b, d and e contained *GGPPS* genes from gymnosperms and angiosperms but not from algae, moss and fern, indicating that these groups might be evolved after separation of algae, ferns and moss. However, *GGPPS* genes from *G. hirsutum* were present in almost all groups except GGPPS-a. Furthermore, the increase in the predicted number of *GGPPSs* in *G. hirsutum* than *G. arboreum* and *G. raimondii* demonstrated duplication events during polyploidization*.* Moreover, the *GGPPS* genes from cotton showed close relationship with cacao *GGPPS* genes in phylogenetic tree, as well as their number and distribution differ in all groups (Fig. 1). For instance, in group GGPPS-c, *GhGGPPS4, GhGGPPS17, GbGGPPS18, GrGGPPS3, GrGGPPS4, GaGGPPS3, GhGGPPS5,* and *GbGGPPS4*, genes showed a close relationship with three cacao genes (*TcGGPPS2, TcGGPPS4* and *TcGGPPS3*), supporting the hypothesis that cacao and cotton were closely related and probably derived from the same ancestors [24]. Additionally, the results of phylogenetic analysis were also verified by constructing another phylogenetic tree using ME (Maximum evolution) method (Additional file 6:Fig. S1). Both phylogenetic trees displayed highly similar results.

**Fig. 2:**
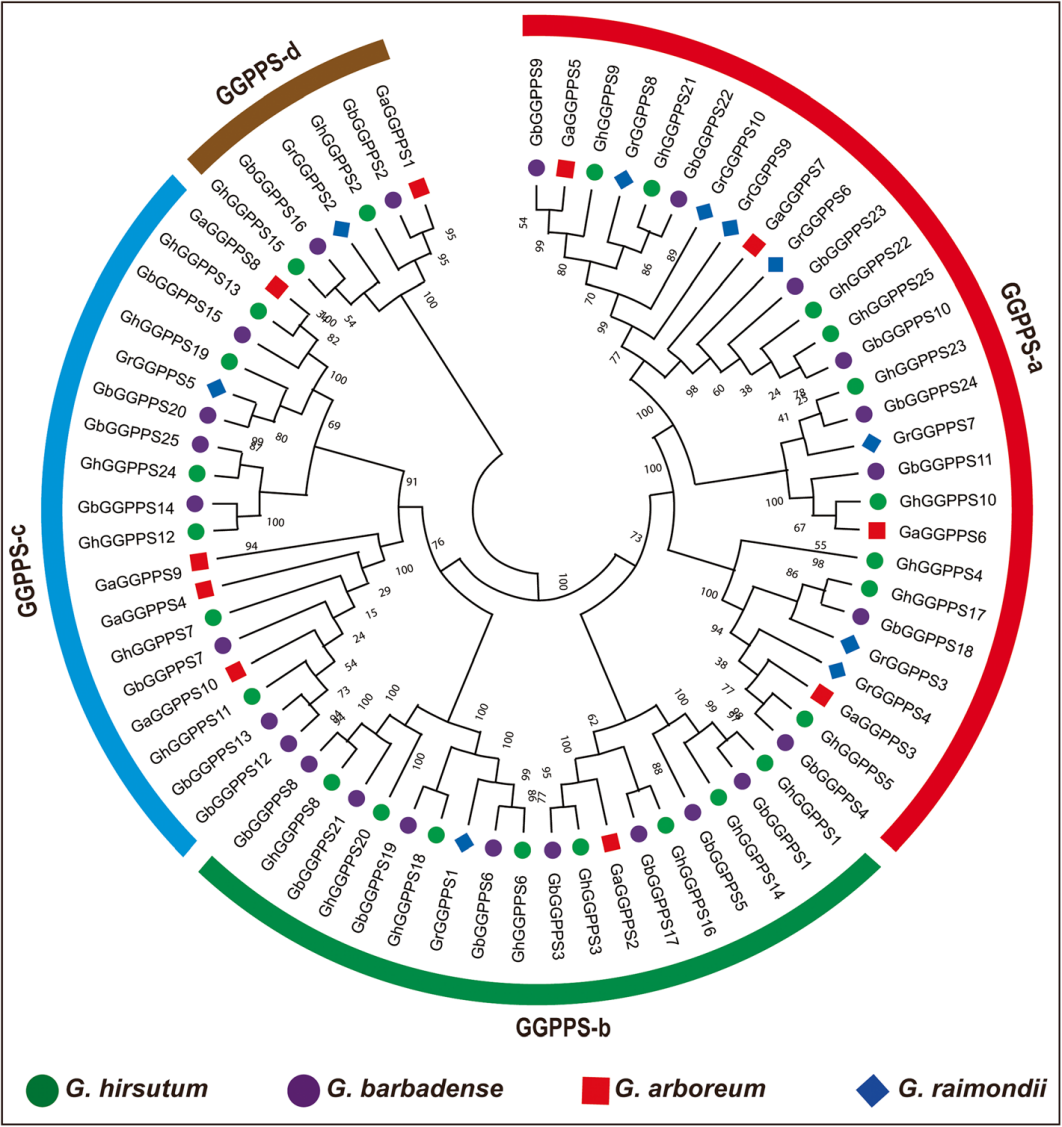
Phylogenetic analysis of *GGPPS* genes in *G. arboreum*, *G. barbadense, G. hirsutum*, and *G. raimondii*. The different color indicated different groups of *GGPPS* gene family

Next, we generated another phylogenetic tree among four cotton species by NJ (neighbor-joining) method. Phylogenetic tree divided 70 cotton *GGPPS* genes into four groups from GGPPS-a to GGPPS-d (Fig. 2). Group GGPPS-a was the biggest group with 28 members while group GGPPS-d was the smallest group with six members of *GGPPS* genes. Out of 70 cotton *GGPPS* genes, six *GGPPS* genes (*GaGGPPS1, GbGGPPS2, GhGGPPS2, GrGGPPS2, GbGGPPS16,* and *GhGGPPS15,*) form a separate group in the phylogenetic tree indicating that *GbGGPPS2, GbGGPPS16, GhGGPPS2* and *GhGGPPS15* are the orthologs of *GaGGPPS1* and *GrGGPPS2* and demonstrating that *GbGGPPS2, GbGGPPS16, GhGGPPS2* and *GhGGPPS15* might be evolved as the result of hybridization of *GaGGPPS1* and *GrGGPPS2* during evolution. However, *GhGGPPS* genes form three groups with GGPPS-a as a largest group (11 genes) followed by GGPPS-c (four genes) and GGPPS-b (ten genes), when another phylogenetic tree (NJ) was constructed among them (Additional file 7: Fig. S2).

### Biophysical properties and sequence logos of GGPPS in *G. hirsutum*

To explore the important characteristics of 25 members of *GGPPS* gene family in *G. hirsutum,* chemical and biophysical properties were predicted including chromosomal position (start and end points), gene length (bp), coding sequence (CDS), protein length (aa), molecular weight (MW), isoelectric point (pI), and grand average of hydropathicity (GRAVY). The gene length ranged from 620 to 6749 bp. Eleven genes with no introns exhibited the lower gene length ranged from 620 to 1118 bp, While two genes (*GhGGPPS6* and *GhGGPPS18*) with 12 numbers of introns showed maximum gene length (6749 bp and 6657 bp) compared with others (Additional file 2: Table S2) indicating that increase in number of introns increases gene length. *GhGGPPS14* and *GhGGPPS20* as the most shortest and longest genes respectively, their coding sequence (CDS), numbers of amino acids (aa), and molecular weights ranged from 621 to 1932 bp, 206–643 aa, 22,741–71,107 Da respectively. The values of isoelectric point of *GhGGPPS* genes ranged from 4.67–9.68. Two proteins GhGGPPS7 (8.5) and GhGGPPS11 (9.68) showed isoelectric point more than 7, indicating that they were alkaline proteins, while all others showed isoelectric point below 7 indicating that they all were acidic proteins. In addition, the average hydropathy value of each residue present in the sequences was calculated to evaluate the GRAVY (Grand Average of Hydropathicity) values of the proteins. The positive GRAVY values of the proteins revealed hydrophobicity, however negative scores revealed hydrophilicity. According to the GRAVY values 14 GhGGPPS proteins were hydrophilic while 11 were hydrophobic (Additional file 2: Table S2). Subcellular localization prediction indicated that 12 GhGGPPS proteins were located in chloroplast, six in cytoplasm, two in mitochondrial, two in plasma membrane and three in nucleus*.*

**Fig. 3:**
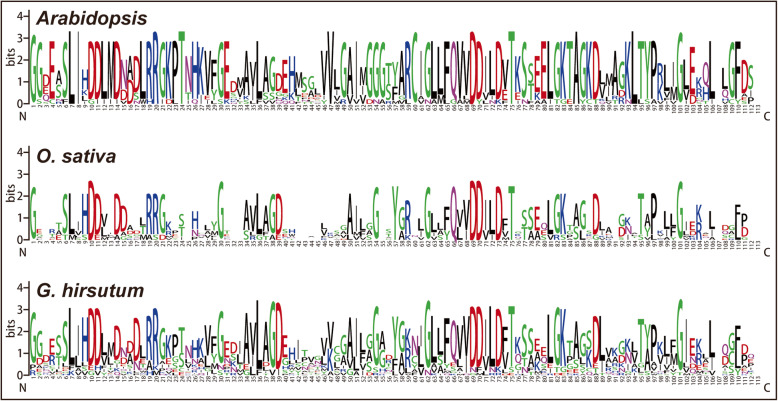
Sequence logos of *Arabidopsis, O. sativa*, and *G. hirsutum*. The N-terminal and C-terminal of *GGPPS* gene domain are indicated by using ‘N’ and ‘C’

Furthermore, the GGPPS protein sequence logos could assist to discover and evaluate the pattern of GGPPS protein sequence conservation in other plant species, as sequence logos provide a more precise description of sequence similarity than consensus sequences. We generated the sequence logos of the conserved amino acid residues in *Arabidopsis,* rice and *G. hirsutum* to check whether the GGPPS family proteins were conserved in monocots and dicots during evolution (Fig. 3). Conserved amino acid residues analysis showed that the sequence logos among monocots and dicots are highly conserved across the N and C terminals.

### Intron/exon structure, protein motifs and promoter *cis*-elements of *GhGGPPS* genes

To further clarify the evolutionary relationships, gene structure and conserved motifs analysis of 25 *GhGGPPS* genes was performed (Additional file 8: Fig. S3a). The phylogenetic tree was build for gene structure and conserved motifs distribution analysis, which divided the *G. hirsutum GGPPS* genes into three groups on the basis of topology (bootstrap value and branch length) of phylogenetic tree. Results indicated that 10 conserved protein motifs were randomly distributed among 25 *GhGGPPS* genes (Additional file 8: Fig. S3b). Most of the GhGGPPS proteins displayed similar motifs distribution pattern, suggesting they might have conserved functions. Motif 4 was present in almost all proteins except three (GhGGPPS25, GhGGPPS15, and GhGGPPS2) GhGGPPS proteins. Motif 10 was identified only in seven proteins (GhGGPPS8, GhGGPPS20, GhGGPPS6, GhGGPPS18, GhGGPPS7, GhGGPPS12, and GhGGPPS24) of group GGPP-b, however it was not identified in proteins of groups GGPP-a and GGPP-c. Comparing to motif 10, motif 7 was not identified in group GGPP-b but in almost all proteins of groups GGPP-a and GGPP-c.

Gene structural analysis indicated that the coding regions of all *GhGGPPS* genes were interrupted by 1–12 introns. Accounting 44% (11*GhGGPPS* genes) of the total *GhGGPPS* genes, lack introns, and 8% (two *GhGGPPS* genes) had maximum number (12 introns) of introns. Two *GhGGPPS* genes (*GhGGPPS3* and *GhGGPPS16*) were interrupted by only one intron. However, some variations of exon and intron sizes were observed between *GhGGPPS* gene family. In most cases, *GhGGPPS* genes within the same group exhibited similar gene structures in regard to the distribution patterns, number and length of introns/exons (Additional file 8: Fig. S3c).

**Fig. 4:**
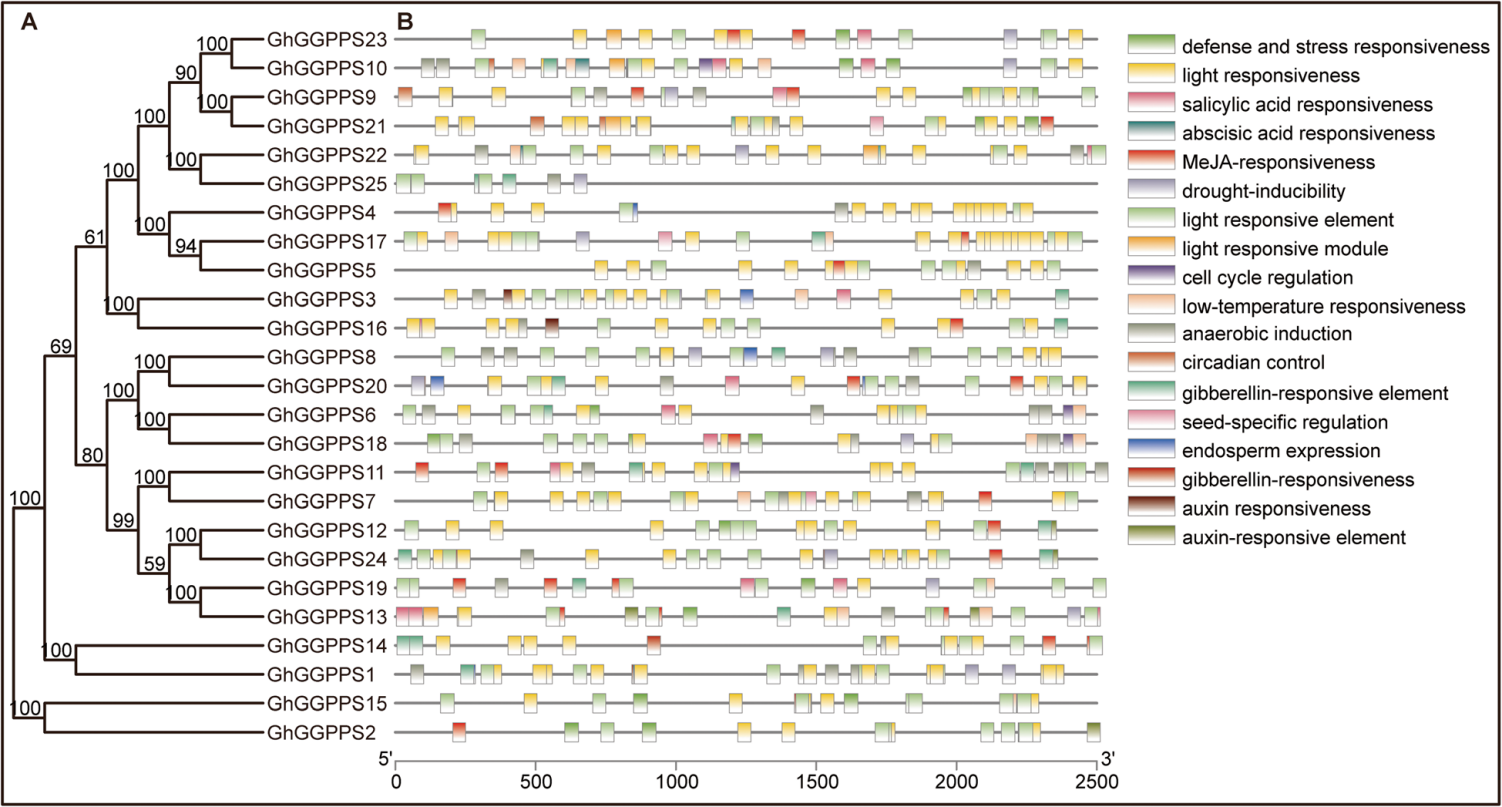
Promoter *cis*-elements analysis of *GhGGPPS* genes. **a** Phylogenetic tree of 25 members of *GhGGPPS* gene family. **b** *GhGGPPS* promoter *cis*-elements distribution and list of names of promoter *cis*-elements with different colors was given to recognize different promoter *cis-*elements

To analyze the possible roles of *GhGGPPS* genes in response to various conditions, promoters of candidate *GhGGPPS* genes were identified and searched for *cis*-elements. The *GhGGPPS* genes promoters shared light responsive boxes and stress-related boxes. Additionally, hormones-related *cis*-elements including MeJA, salicylic acid, gibberellin, auxin, and abscisic acid were also found in the *GhGGPPS* genes promoters (Fig. 4). Based on the results, we observed that stressed and hormones-related *cis*-elements were existed in almost all *GhGGPPS* genes. Some of the gene promoter regions contained various elements for plant growth and development including circadian, endosperm expression, cell cycle regulation, and seed specific regulation. The identified motifs showed that *GhGGPPS* genes may be regulated by various *cis*-elements within the promoter during growth and development.

### Chromosomal location, colinearity analysis and gene duplication of *GhGGPPS*

Chromosomal location of *GhGGPPS* genes were investigated on their corresponding chromosomes (At and Dt sub-genome chromosomes of *G. hirsutum*). The chromosomes distribution indicated that 23 genes out of 25 were unevenly distributed among 12 chromosomes while two genes (*GhGGPPS11* and *GhGGPPS25*) were assigned to scaffolds. Uneven distribution of *GhGGPPS* genes on chromosomes suggested that genetic variation existed in the evolutionary process. Six chromosomes A01, A07, A08, A10, A11, and A13 from At sub-genome contained 12 genes and six chromosomes D01, D07, D09, D10, D11, and D13 from Dt sub-genome contained 11 genes (Additional file 9: Fig. S4). However there was no gene located on chromosome nine (A09) of At sub-genome as well as chromosome eight (D08) of Dt sub-genome. Six *GhGGPPS* genes *GhGGPPS6, GhGGPPS7, GhGGPPS10, GhGGPPS18, GhGGPPS19,* and *GhGGPPS24* were located on six chromosomes of At /Dt sub-genome such as A07, A08, A11, D07, D09, and D13 respectively. Chromosomes A01 and D01from At /Dt sub-genomes have a higher number of *GhGGPPS* genes as compared to others.

**Fig. 5:**
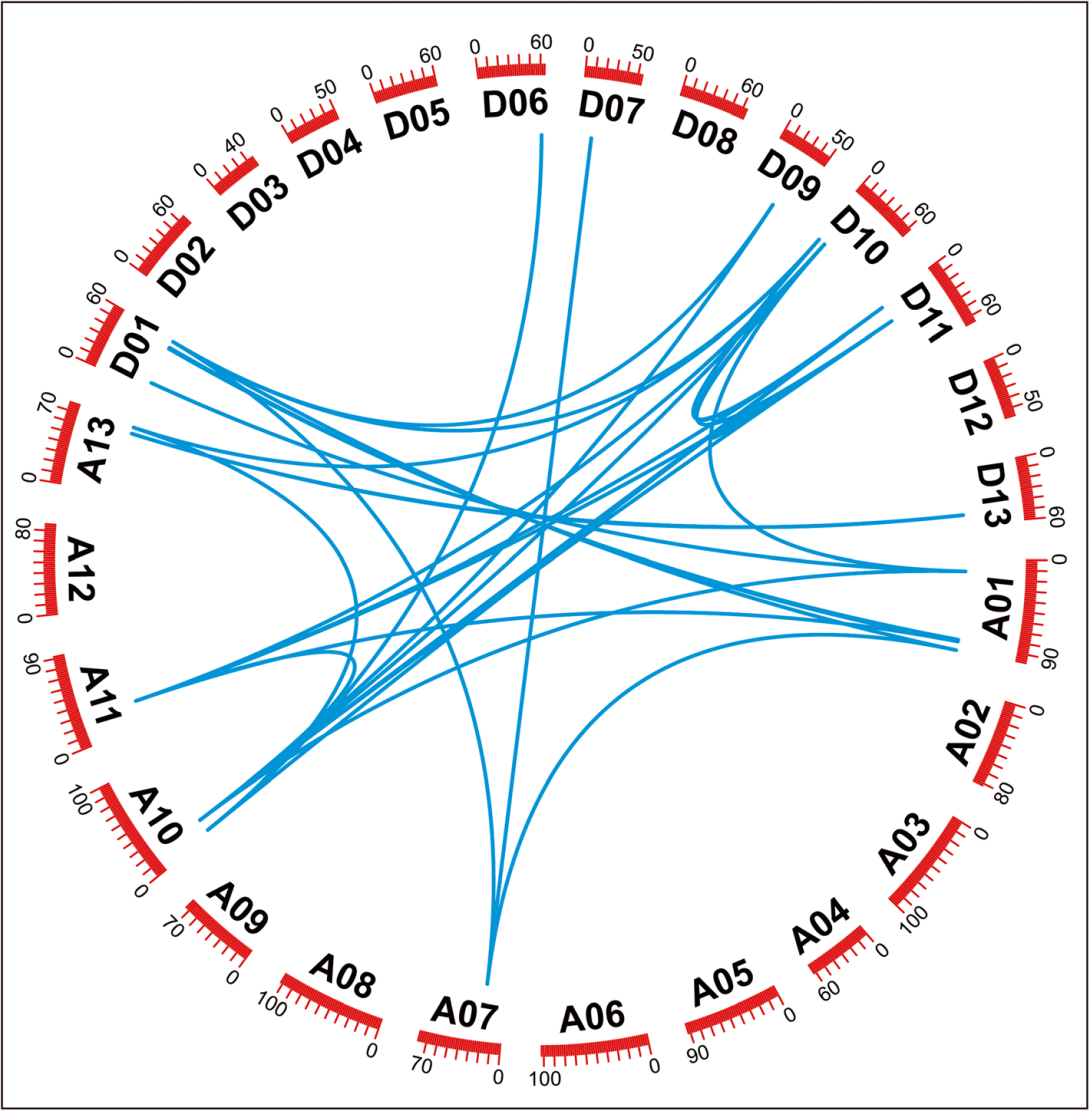
Collinearity analysis of *GhGGPPS* genes. A01-A13 represented *At* sub-genomes chromosomes while D01-D13 represented *Dt* sub-genomes chromosome. The orthologous/paralogous gene pairs were specifying by blue color lines

Collinearity analysis of *GhGGPPS* gene family indicated that there was 29 pairs of orthologous/paralogous *GhGGPPS* genes in *G. hirsutum* and that most of *GhGGPPS* genes loci were conserved for both sub-genomes (At and Dt) (Additional file 3: Table S3, Fig. 5). Tandem and segmental duplication events are the main causes of gene-family expansion in *G. hirsutum*. To understand the gene duplication event within the *G. hirsutum* genome, we determined the tandem and segmental duplication during the evolution of *GGPPS* gene family here. According to whole genome duplication analysis, it is observed that 22 pairs of *GhGGPPS* genes experienced segmental duplication while one tandem and two were dispersed duplication events, which suggested that segmental duplication contributed mainly in the expansion of the *GGPPS* family members in *G. hirsutum* genome (Additional file 3: Table S3).

### Tissues specific expression of *GhGGPPS* genes

Spatiotemporal expression of transcript is mostly correlated with the biological function of gene. To investigate the tissue specific expression patterns of different *GhGGPPS* genes, RNA-seq data were downloaded from NCBI to generate heat map (Additional file 4: Table S4). We noted that all the genes were clustered according to their expression patterns in the vegetative organs (root, stem, and leaf), reproductive organs (torus, petal, stamen, pistil, and calycle), ovules (− 3, − 1, 0, 1, 3, 5, 10, 20, 25 and 35 DPA) and fibers (5, 10, 20, and 25 DPA) (Additional file 10: Fig. S5). According to the heat map all genes exhibited ubiquitous expression with no specific pattern. However, six genes (*GhGGPPS2, GhGGPPS15, GhGGPPS8, GhGGPPS22, GhGGPPS3,* and *GhGGPPS16*) showed higher expression in almost all vegetative, reproductive, ovule, and fiber tissues. In contrast, seven genes (*GhGGPPS11, GhGGPPS19, GhGGPPS5, GhGGPPS4, GhGGPPS17, GhGGPPS7*, and *GhGGPPS14*) showed very low expression in vegetative, reproductive, ovule and fiber tissues indicating that these genes were pseudo genes or could function with low transcripts in cotton development.

**Fig. 6:**
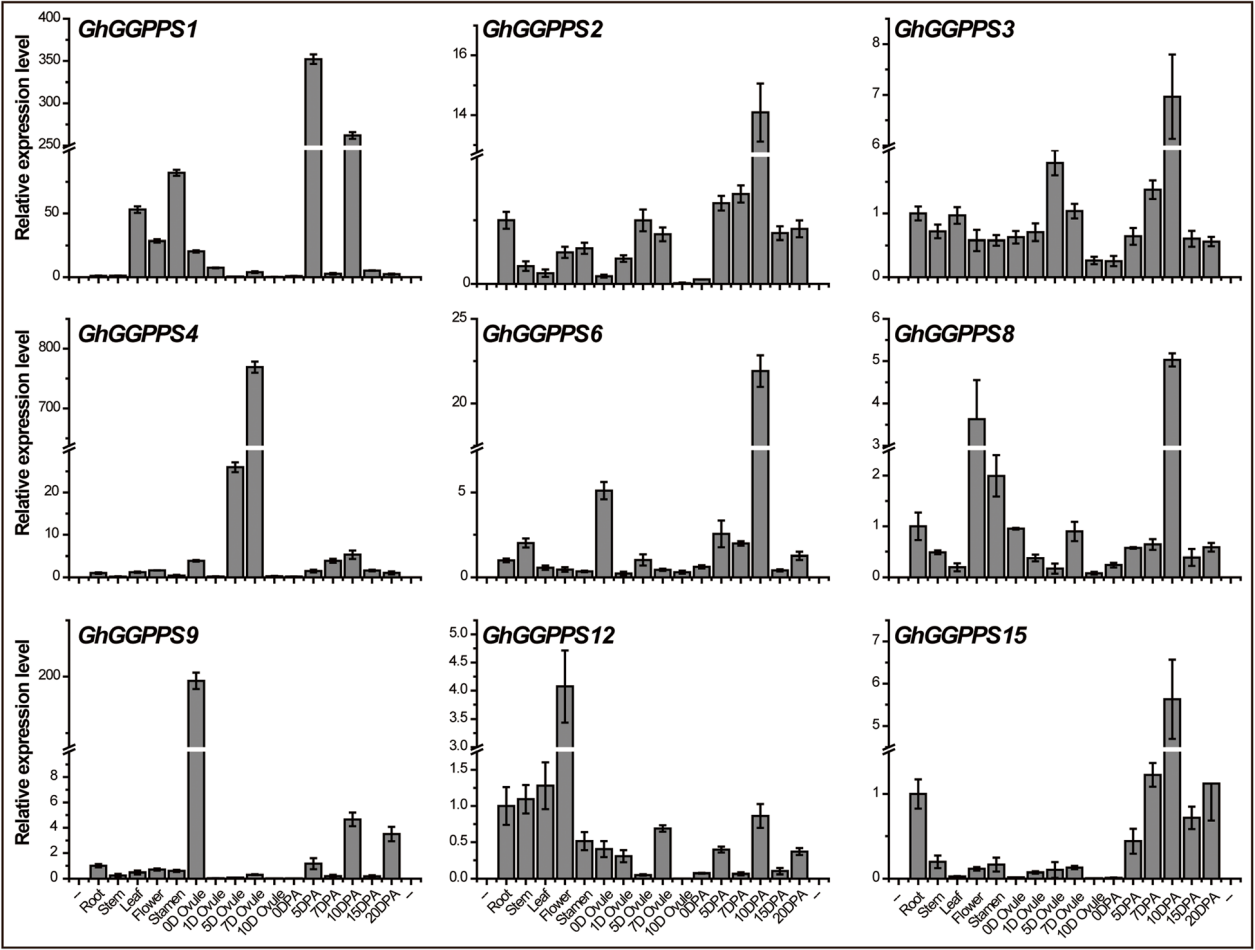
Expression patterns of *GhGGPS*genes in different tissues of *G. hirsutum*. qRT-PCR analysis was performed for nine segmentally duplicated *GhGGPPS* genes. Plant tissues were collected at different developmental stages such as root, stem, leaf, flower, stamen, 0, 1, 5, 7, and 10 DPA ovule tissues as well as 0, 5,7, 10, 15, and 20 DPA fiber tissues

The different members of the same gene family can play different physiological functions by exhibiting their expression in different tissues/organs [25]. In earlier study, different expression pattern of *GGPPS* genes was observed in different organs and seedlings of *A. thaliana* [4]. To elucidate the roles of *GhGGPPS* genes in different tissues of upland cotton, 9 segmentally duplicated *GhGGPPS* genes were selected for qRT-PCR. Transcript level of *GhGGPP4* and *GhGGPPS9* showed significant and specific higher in primary developmental ovules from 0 to 7 DPA, indicating their potential roles in earlier development of ovule and the fiber cell initiation. Others like *GhGGPPS1, GhGGPPS6* and *GhGGPPS15* showed higher expression in later development stages of fiber, indicating that they might have important participation in fiber elongation and secondary cell deposition. *GhGGPPS2, GhGGPPS3* and *GhGGPPS8* showed conserved expression in almost all tissues, indicating they may play some conserved and basic roles in plant different development stages. Whereas expression profiles in non-reproductive tissues of *GhGGPPS12* indicated its potential roles in vegetative development (Fig. 6).

### Expression pattern of *GhGGPPS* gene family under abiotic and hormonal stresses

**Fig. 7:**
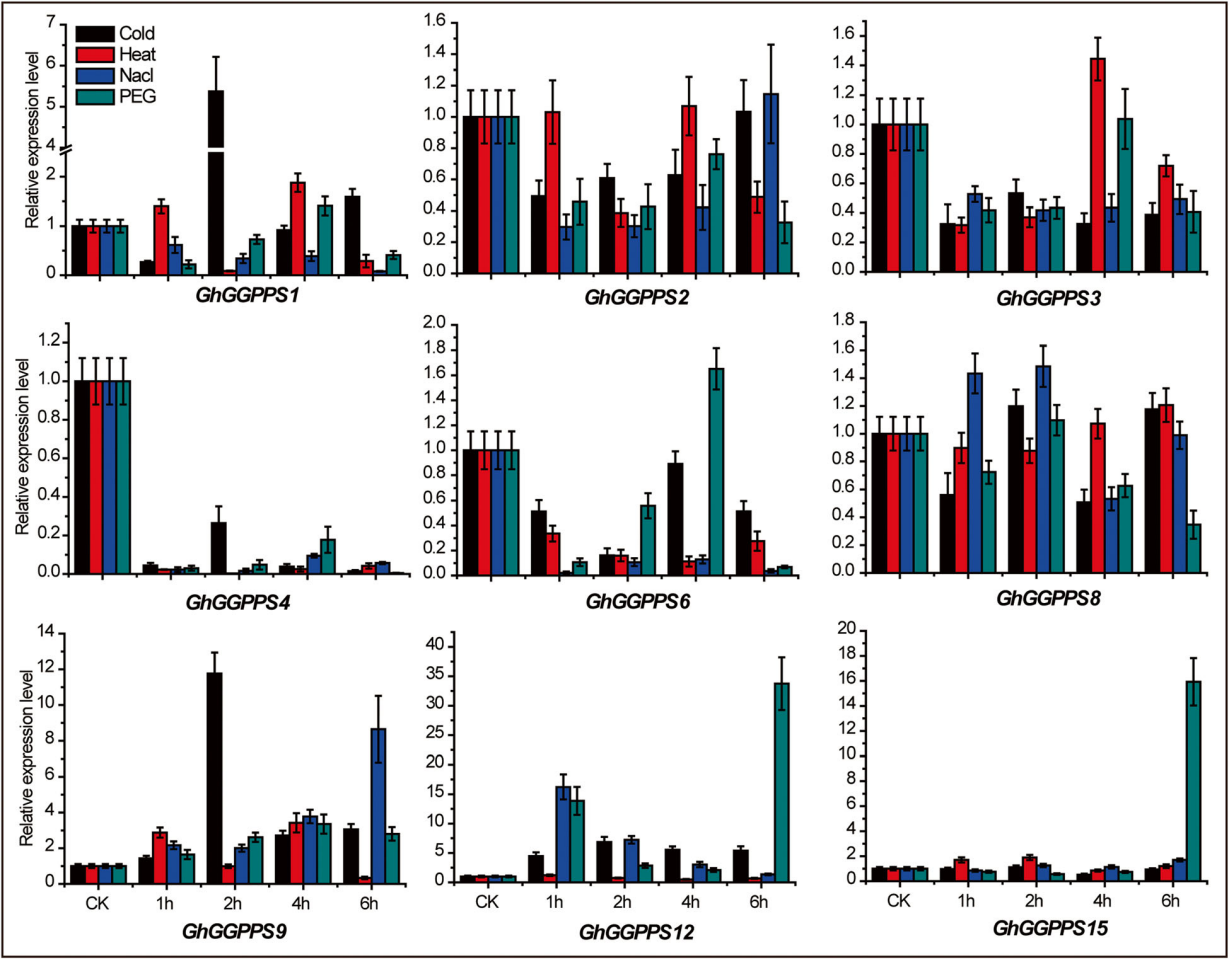
Expression patterns of *GhGGPPS* genes under various abiotic stresses at different time points. The relative expression was normalized to the CK (0 h) whereas error bars represented the standard deviation (SD) of three replicates

To further investigate the physiological and functional significance of *GhGGPPS* genes, we investigated the expression patterns of *GhGGPPS* genes under different stresses such as Cold, NaCl, PEG, and heat and hormonal treatments such as brassinolide (BL), gibberellins (GA), indole-3-acetic acid (IAA), salicylic acid (SA) and methyl jasmonate (MeJA). Firstly, the expression pattern of *GhGGPPS* genes under abiotic stresses were analyzed by RNA-seq data downloaded from NCBI, and a heat map was generated. Results depicted that the expression of *GhGGPPS* genes were up or down-regulated under different treatments and that all the genes were clustered according to their different responses (Additional file 11: Fig. S6). *GGPPS10, GGPPS23, GGPPS12, GGPPS24, GGPPS9, GGPPS3,* and *GGPPS22* were up-regulated with almost all abiotic stresses. To confirm that, qRT-PCR was performed for 9 selected *GhGGPPS* genes under different abiotic stresses including Cold, NaCl, PEG, and heat. qRT-PCR results revealed that *GhGGPPS1, GhGGPPS9,* and *GhGGPPS8* responded to almost all abiotic stresses. Whereas, the transcript level of *GhGGPPS1* and *GhGGPPS9* was up-regulated about 5 and 11 folds under 2 h cold stress respectively than that of control, while the transcript level of *GhGGPPS8* was up-regulated 0.5 folds under 1 and 2 h NaCl stress suggesting that these genes might play an important positive role in cold and NaCl stresses. But the 33 and 15 folds up-regulated transcript level of *GhGGPPS12* and *GhGGPPS15* respectively were perceived at 6 h drought stress, indicating their specific and positive role in drought stress. Interestingly, the expression of *GhGGPPS4* was down-regulated to no more than 30% of the transcripts in mock in response to all given abiotic stresses, as well as expression of *GhGGPPS3* was down-regulated to about half level comparing to mock, indicating that both *GhGGPPS4* and *GhGGPPS3* are negative regulators of abiotic stresses in cotton (Fig. 7).

**Fig. 8:**
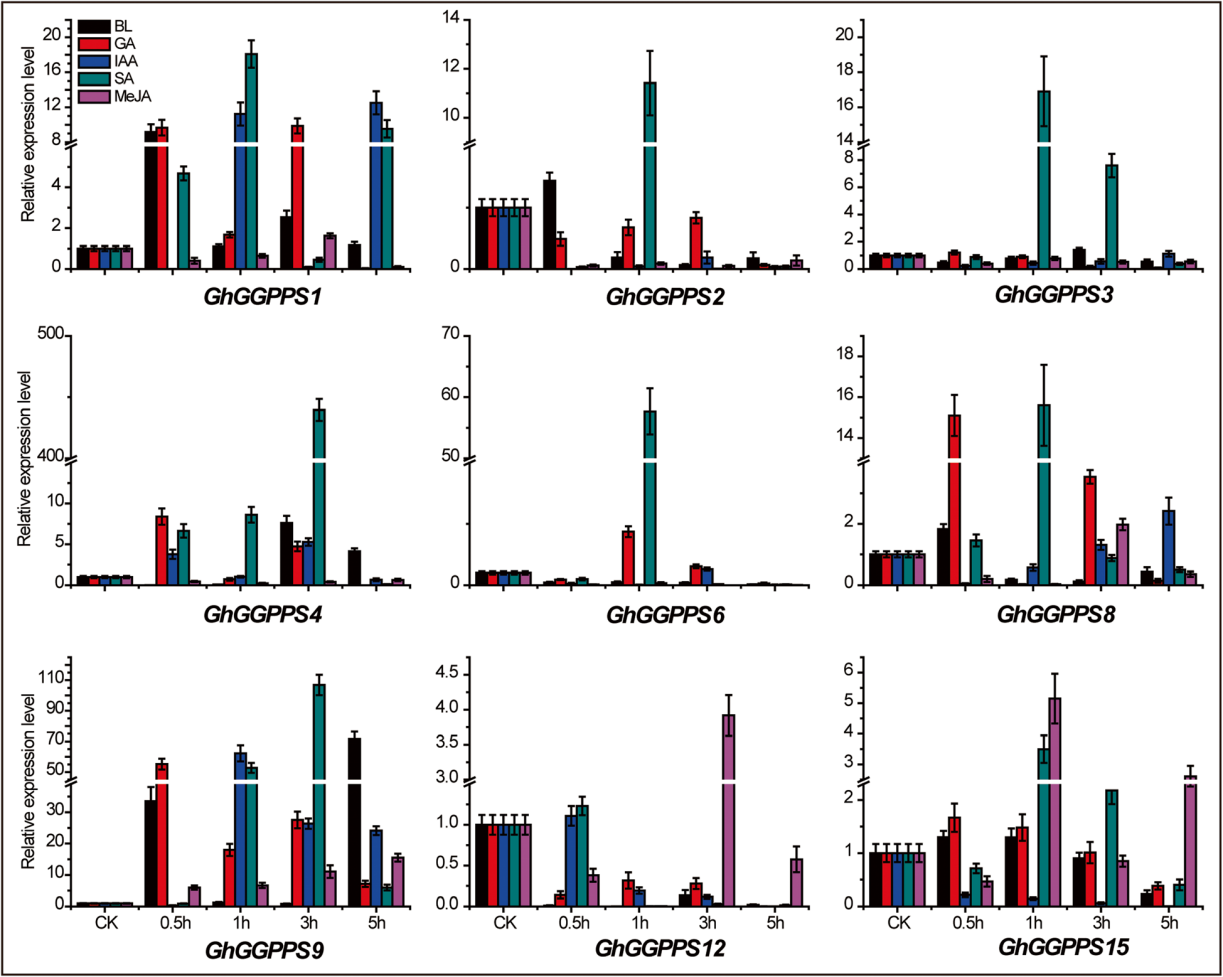
Relative expression patterns of *GhGGPPS* genes with five hormonal (BL, GA, IAA, SA, and MeJA) stresses at different time points

Secondly, we investigated the expression patterns of these genes under different hormonal treatments (BL, GA, IAA, SA and MeJA). qRT-PCR results indicated that *GhGGPPS1, GhGGPPS4, GhGGPPS9,* and *GhGGPPS15* were up-regulated with all hormonal stresses with different time point, demonstrating that these genes are involved in all five hormonal signaling pathways (Fig. 8). *GhGGPPS1, GhGGPPS2, GhGGPPS3, GhGGPPS6,* and *GhGGPPS8* were preferentially up-regulated with 1 h SA and 0.5 h GA treatments. In addition, the up-regulated expression of *GhGGPPS4* and *GhGGPPS9* was observed when subjected to 3 h SA stress. The up-regulated transcript level of *GhGGPPS12* and *GhGGPPS15* were detected at 3 and 1 h after MeJA treatment respectively, proposed that these two genes are important for MeJA signaling.

## Discussion

Most of the essential plant isoprenoids are derived from allylicprenyl diphosphates farnesyl-PP (FPP) and geranylgeranyl-PP (GGPP) [26]. GGPP is essential for primary and secondary isoprenoid compounds synthesis, however complete characterization of the *GGPPS* gene family was reported only in *A. thaliana* [4]. To fully understand the role of *GhGGPPS* isozymes in *G. hirsutum*, a complete characterization of this type of enzyme class was crucial. *G. hirsutum* is one of the most widely cultivated cotton specie that accounts for more than 90% of the world cotton fiber yield [27]. In this study, we analyzed the evolutionary relationships of *GGPPS* gene family in 18 plant species and characterized the biophysical properties, chromosomal location, gene duplication, selection pressure and expression in different plant tissues and responses to various abiotic and hormone stresses in *G. hirsutum GGPPS* gene family. The *GGPPS* genes in allotetraploid cotton *(G. hirsutum)* were focused to understand the roles of *GGPPS* gene family in cotton development.

### Evolutionary characteristic of *GGPPS* gene family

During the identification of *GGPPS* gene family members in different plant species, only one *GGPPS* gene was identified in algae indicating that the plant-specific *GGPPS* genes might have originated from a common ancestor of land plants green algae (Additional file 5:Table S5). Interestingly, the number of *GGPPS* genes identified in *G. hirsutum* were more than double of the *GGPPS* genes identified in *G. arboreum* and *G. raimondii,* possibly because of its formation as an allotetraploid following hybridization of A and D genome progenitors [28, 29]. Phylogenetic tree divided the 159 *GGPPS* genes into five groups GGPPS-a to GGPPS-e, where group GGPPS-c was the biggest group while GGPPS-e was the smallest group with minimum number of *GGPPS* genes. Group GGPPS-c contained *GGPPS* genes from 14 species out of 18 along with one *GGPPS* gene from first land plant algae *(C. reinhardtii)* indicating that it was the oldest group evolved from the common ancestor and that *GGPPS* genes might have originated from a common ancestor of land plants as proved in previous studies [22, 23]. These findings were also supported by the analysis of conserved amino acid residues of *G. hirsutum, O. sativa* and *A. thaliana*. These results demonstrated that sequence logos were significantly conserved across N and C terminals of monocotyledons and dicotyledons plant species, exhibiting that *GGPPS* gene family remained conserved during evolutionary process. Moreover, the phylogenetic tree revealed that *GGPPS* genes from four cotton species showed more close relationship with cacao genes and predicted that cotton and cacao are evolved from common ancestors as proved from previous studies [24]. Additionally, NJ tree validation by ME tree showed the similar results including number of groups as well as positions of genes in groups.

To enhance the understanding of *GGPPS* gene family diversification during the history of evolution and domestication, a phylogenetic analysis was performed among *G. barbadense, G. arboreum, G. raimondii,* and *G. hirsutum.* Phylogenetic tree represented that group GGPP-a was the biggest group while GGPPS-d was the smallest. Group GGPPS-d had six *GGPPS* genes (*GaGGPPS1, GbGGPPS2, GhGGPPS2, GrGGPPS2, GbGGPPS16,* and *GhGGPPS15,*), one from *G. arboreum* and *G. raimondii* while two from *G. hirsutum* and *G. barbadense* indicating that *GbGGPPS2, GbGGPPS16, GhGGPPS2* and *GhGGPPS15* are the orthologs of *GaGGPPS1* and *GrGGPPS2*. These results also represented that *GbGGPPS2, GbGGPPS16, GhGGPPS2* and *GhGGPPS15* might be evolved from the hybridization of *GaGGPPS1* and *GrGGPPS2* and further supported that *G. arboreum* and *G. raimondii* is the donor species of A-subgenome and D-subgenome, respectively. To understand the evolution relationship in detail, a phylogenetic tree among *G. hirsutum GGPPS* genes was constructed and it divided the 25 *GhGGPPS* genes into three groups. The *GhGGPPS* genes showing close evolutionary relationship were clustered together in phylogenetic tree, suggesting that they might play similar functions in plant growth and development.

Deeper analysis of *GhGGPPS* genes indicated that gene length of *GhGGPPS* genes increased with the increase of introns/gene ranging from 0 to 12 introns/genes with the gene length of 620–6749 bp. Further, it has been found that *GhGGPPS* belonging to the same group share similar exon-intron structures and protein motif distribution pattern along with some conserved motifs, indicated that *GhGGPPS* gene family is more conserved. Here, we also speculated that the encoded proteins with similar motifs might be associated with particular functions related to growth, development and stress tolerance in cotton. *GhGGPPS20* is the largest presumed protein (71,107 Da), while *GhGGPPS14* is the gene with smallest molecular weight (22,741 Da). Further, 14 GhGGPPS proteins were hydrophilic while11 proteins were hydrophobic and they were found to be localized in different cell organelles, such as chloroplast, mitochondria, plasma membrane and nucleus.

### Gene duplications among *GhGGPPS* genes

Orthologs are genes that were derived from the same ancestral gene; encode proteins with similar biological functions, whereas paralogs are from a single gene as a result of duplication event, encode proteins with different functions [30, 31]. It is found that duplicated genes are mostly involved in the formation of paralogous genes present in gene families. Uneven gene distribution of *GhGGPPS* genes on the chromosomes of At and Dt sub-genome indicated that *GhGGPPS* genes experienced gene duplication events during evolution and hybridization. The At and Dt sub-genome donors (*G. arboreum* and *G. raimondii*, respectively) of upland cotton are close relatives sharing the same number of orthologs and doubling numbers of *GGPPS* genes in upland cotton. In this study, 70 identified *GGPPS* genes in four representative cotton species were used to further explore the evolution of the *GGPPS* family.

Additionally, colinearity analysis exhibited that 29 pairs of orthologous/paralogous *GhGGPPS* genes were identified in present study as a result of gene duplication. Deeper investigations showed that 22 (88%) *GhGGPPS* genes out of 25 originated from segmental duplication and 1 gene (4%) originated from tandem duplication; revealed high segmental and low tandem duplication events in *GhGGPPS* genes. Many gene families underwent segmental duplication and attributed the gene family expansion and functional divergence in cotton [32–35]. Here, both tandem and segmental duplications contributed to the expansion of *GhGPPS* gene family, but the segmental duplication might play a more critical role.

### Expression profile analysis of *GhGGPPS* genes

Gene expressional patterns explain the functions of that gene in plant growth and development [36]. Our results indicated that promoter regions of *GhGGPPS* contained various elements related to plant growth and development including circadian, endosperm expression, cell cycle regulation, and seed specific regulation. Further, various *cis*-elements for different stress responses such as hormones (SA, ABA, GAs, auxin, and MeJA) and abiotic stress (low temperature and drought) were also identified. *Cis*-elements prediction results suggested that *GhGGPPS* genes might play important roles during stresses tolerance and in plant growth and development. So, to investigate the possible functions of *GhGGPPS* genes in cotton under different stresses and development, we analyzed the transcript level of nine selected *GhGGPPS* genes by qRT-PCR. The *GhGGPPS* genes showed different expression patterns, and the particular and up-regulated expression in ovule and fiber of some *GhGGPPS* (e.g. *GhGGPPS4, GhGGPPS9,* and *GhGGPPS15*) genes indicated the important roles of these genes in ovule and fiber development (Fig. 6).

Previously, it has been reported that *GGPPS* genes functions were related to plant development, but the function of *GGPPS* genes under different stresses has not been reported yet. Thus, to find whether they might play some roles in stress response, the responses of *GhGGPPS* genes expression under various abiotic and hormonal treatments were determined. It was observed that most of the *GhGGPPS* genes were induced by different abiotic stresses, such as *GhGGPPS1* and *GhGGPPS9* were up-regulated under 2 h cold, *GhGGPPS3* was up-regulated with 4 h heat, *GhGGPPS8* was up-regulated under 1 and 2 h NaCl, and *GhGGPPS15* was up-regulated by 6 h PEG treatment, indicating that *GhGGPPS* genes might positively participate in abiotic stress responses. However, the down-regulation of *GhGGPPS4* in all the abiotic stresses indicated which may be very good target to improve the broad-spectrum abiotic tolerance of cotton by CRISPR-CAS9 gene editing technology. Next, four *GhGGPPS* genes (*GhGGPPS1, GhGGPPS4, GhGGPPS9,* and *GhGGPPS15*) were up-regulated with all hormonal stresses, whereas eight genes (*GhGGPPS1, GhGGPPS2, GhGGPPS3, GhGGPPS4, GhGGPPS6, GhGGPPS8, GhGGPPS9* and *GhGGPPS15)* were preferentially induced with SA stress at different time point. The up-regulated expression levels of two genes (*GhGGPPS12* and *GhGGPPS15*) were detected at 3 and 1 h MeJA stress treatments, demonstrating the positive participation of *GhGGPPS* genes under hormonal stresses and their crucial roles in hormone signaling pathways.

## Conclusions

Present work represents a genome-wide characterization and expression analysis of the *GGPPS* gene family in *G. hirsutum*. Segmental duplication was found as an important source for the expansion of the *GGPPS* gene family in cotton. Biophysical properties indicated that *GhGGPPS* genes localized to different cellular compartments, which suggested that the enzymes are associated with specific functions. Based on the abundance and spatiotemporal expression patterns of *GhGGPPS* genes transcripts, *GhGGPPS* genes showed associated with ovule and fiber development, and regulated by abiotic and hormonal stresses. Such as *GhGGPPS4, GhGGPPS9* and *GhGGPPS15* may be utilized well in the fiber yield and quality improvement, while *GhGGPPS4* may be also a potential target of gene editing to enhance the plant abiotic tresses tolerance. The results provide useful information for further study related to structure, function and phylogenetic relationships of these gene family members and are helpful for the determination of key genes to improve stress tolerance and developmental research in cotton and other valuable plants.

## Methods

### Sequence identification

The *AtGGPPS* genes were used as queries to identify the *GGPPS* genes in other 17 plant species including *O. sativa* (version 10.0)*, S. bicolor* (version 10.0), *G. max* (version 10.0)*, S. moellendorfii* (version 1.0)*, P. patens* (version 3.3)*, C. reinhardtii* (version 5.5)*, Z. mays* (version 10.0), *P. taeda* (version 1.0)*, V. vinifera* (version 10.0)*, S. tuberosum* (version 10.0)*, M. truncatula* (version 10.0)*, B. napus* (version 1.0)*, T. cacao* (version 10.0)*, G. arboreum* (ICR, version 1.0)*, G. raimondii* (JGI, version 2.0)*, G. barbadense* (NAU, version 1.1), and *G. hirsutum* (NAU, version 1.1) as described in previous study [37]*.* The *Arabidopsis,* cotton (*G. arboreum, G. hirsutum, G. barbadense* and *G. raimondii*) and other plant species databases were downloaded from TAIR 10 (http://www.arabidopsis.org), COTTONGEN (https://www.cottongen.org/) and Phytozome v11 (https://phytozome.jgi.%20doe.gov/pz/portal) respectively. The GGPPS protein sequences were confirmed by SMART [38], Interproscan 63.0 (http://www.ebi.ac.uk/InterProScan/) [39] and hidden Markov model (HMM) as described previously [32].

Additionally, biophysical properties and subcellular localization was predicted by ExPASy-ProtParam tool (http://us.expasy.org/tools/protparam.html) and softberry (www.softberry.com) respectively.

### Phylogenetic tree and sequence logos analysis

To construct a phylogenetic tree, *GGPPS* genes were aligned using “align by muscle” and tree was constructed using MEGA 7.0 with neighbor-joining (NJ) algorithm and minimum evolution (ME) methods. The 1000 bootstrap replicates with 50% cutoff values were used and tree was then visualized by Tree View 1.6 (http://etetoolkit.org/treeview/).

Next, for sequence logos analysis, GGPPS proteins of *A. thaliana,* rice, and *G. hirsutum* were aligned by Clustal X 2.0 [40] and sequence logos were generated by WEBLOG [41] as described previously [42].

### Intron/exon structure, protein motif distribution and promoter *cis*-elements analysis

*GGPPS* gene structure for all *GhGGPPS* genes was generated by Gene Structure Display Server (GSDS) 2.0 [43]. For protein motifs, MEME program [44] was used as described previously [34]. 2.5-kb upstream sequence regions were used for *GhGGPPS* promoter *cis*-element analysis and *cis*-elements were analyzed in PlantCARE [45].

### Chromosomal distribution, collinearity and transcriptomic data analysis

The position information of *GhGGPPS* genes on the chromosome were acquired from (https://cottonfgd.org/search) and physical locations on chromosomes was visualized by MapInspect (http://www.plantbreeding.wur.nl/UK/software_mapinspect.html) [46]. Colinearity analysis was performed by using methods described previously [47] and data obtained from MCScan was used in CIRCOS [48] to generate the figure.

For expression analysis, online available transcriptomic data (https://www.ncbi.nlm.nih.gov/pmc/articles/PMC4482290/) was used as described in previous studies [32, 34]. TopHat and cufflinks were used for RNA-seq expression analysis and the gene expressions were uniformed in fragments per kilo base million (FPKM) [49]. Genesis software was used to generate the heat map [50] of *GGPPS* genes.

### Plant material, RNA extraction and qRT-PCR analysis

The expression pattern of *GhGGPPS* genes in different tissues such as root, stem, leaf, flower, stamen, 0, 1, 5, 7, and 10 DPA ovule tissues as well as 0, 5, 7, 10, 15, and 20 DPA fiber tissues was estimated. These tissues were collected from ZM24 (also as CCRI24), a national certified specie of China (No. GS08001–1997) which was obtained from the Institute of Cotton Research, the Chinese Academy of Agricultural Sciences and grown under field conditions in Zhengzhou China [51, 52]. Abiotic stresses such as cold (4 °C), heat (38 °C), NaCl (300 nmol L^− 1^), and PEG 6000 (10%) were applied for 1, 2, 4, 6 h with 3-leaf stage seedlings; hormonal treatments including BL (10 μM), GA (100 μM), IAA (100 μM), SA (10 μM) and MeJA (10 μM) were applied at 3-leaf stage for 0, 0.5, 1, 3 and 5 h. All samples RNA was extracted using RNAprep Pure Plant Kit (TIANGEN, Beijing, China) and cDNA was synthesized by Prime Script® RT reagent kit (Takara, Dalian, China). SYBR Premix Ex Taq™ II (Takara) was used for PCR amplifications and each assay contained three repeats. *GhHis3* (Gene Bank, accession number AF024716) was used as an internal control [53] and calculations were carried out as described [54]. Primers used in this study were enlisted in Additional file 5: Table S5.

## Supplementary information

**Additional file 1: Table S1.** Renaming of *GGPPS* genes from 18 species. (ODS 14 kb)

**Additional file 2: Table S2.** Prediction of biophysical properties of 25 *GhGGPPS* genes. (ODS 19 kb)

**Additional file 3: Table S3.** Orthologous/paralogous gene pairs and gene duplication types of *GhGGPPS* gene family. (ODS 14 kb)

**Additional file 4: Table S4.** Transcriptomic data of tissue specific expression and abiotic stress responses of *GhGGPPS* gene family. (ODS 26 kb)

**Additional file 5: Table S5.** List of primers for qRT-PCR. (ODS 13 kb)

**Additional file 6: Figure S1.** Evolutionary relationship among 159 *GGPPS* genes from 18 plant species using maximum evolution method.

**Additional file 7: Figure S2.** Evolutionary relationship of 25 *GGPPS* genes in *G. hirsutum*. Phylogenetic tree was constructed using MEGA software.

**Additional file 8: Figure S3.** The gene structure analysis of *GGPPS* gene family in *G. hirsutum.* (A) The unrooted neighbor-joining (NJ) tree was constructed based on the *GhGGPPS* domains. (B) *GGPPS* gene family conserved protein motifs distribution. To identify different protein motifs of *GhGGPPS* gene family numbers (1–10) and different colors were given. (C) *GhGGPPS* gene family exon–intron structure was obtained by using GSDS 2.0.

**Additional file 9: Figure S4.** The distribution of *GhGGPPS* genes on the chromosomes of *At* and *Dt* sub genome of *G. hirsutum*.

**Additional file 10: Figure S5.** Heat map of *GhGGPPS* genes under different abiotic stresses. The RNA-Seq expression profiles of *G. hirsutum* were used for relative expression levels of *GhGGPPS* genes, gene expression level depicted in different colors on the scale.

**Additional file 11: Figure S6.** Expression levels of *GhGGPPS* genes in 22 tissues of *G. hirsutum.* RNA-Seq expression profiles were used to generate the heat map through Genesis software.

## Data Availability

All data used in this study are included in this article and additional files. Transcriptome data used for gene expression analysis is available in Additional file 4 Table S4 and could be downloaded from https://www.ncbi.nlm.nih.gov/pmc/articles/PMC4482290/. The Genome sequence and annotation datasets that supported our findings are available in: *A. thaliana*: http://www.arabidopsis.org COTTON: https://www.cottongen.org/ Other species: https://jgi.doe.gov/data-and-tools/phytozome/

## Abbreviations

NaCl: Sodium chloride
PEG: Polyethylene glycol
GA: Gibberellic acid
IAA: Indole-3-acetic acid
BL: Brassinolide
MeJA: Methyl jasmonate
SA: Salicylic acid
DPA: Days post-anthesis
μM: Micro-molar
Mya: Million years ago
BLAST: Basic Local Alignment Search Tool
