# Supplementary figures and images for: Genome-wide characterization and expression analysis of geranylgeranyl diphosphate synthase genes in cotton (Gossypium spp.) in plant development and abiotic stresses
Source: BMC Genomics. 2020 Aug 15;21:561. doi: 10.1186/s12864-020-06970-8 (PMC7430837; doi:10.1186/s12864-020-06970-8)

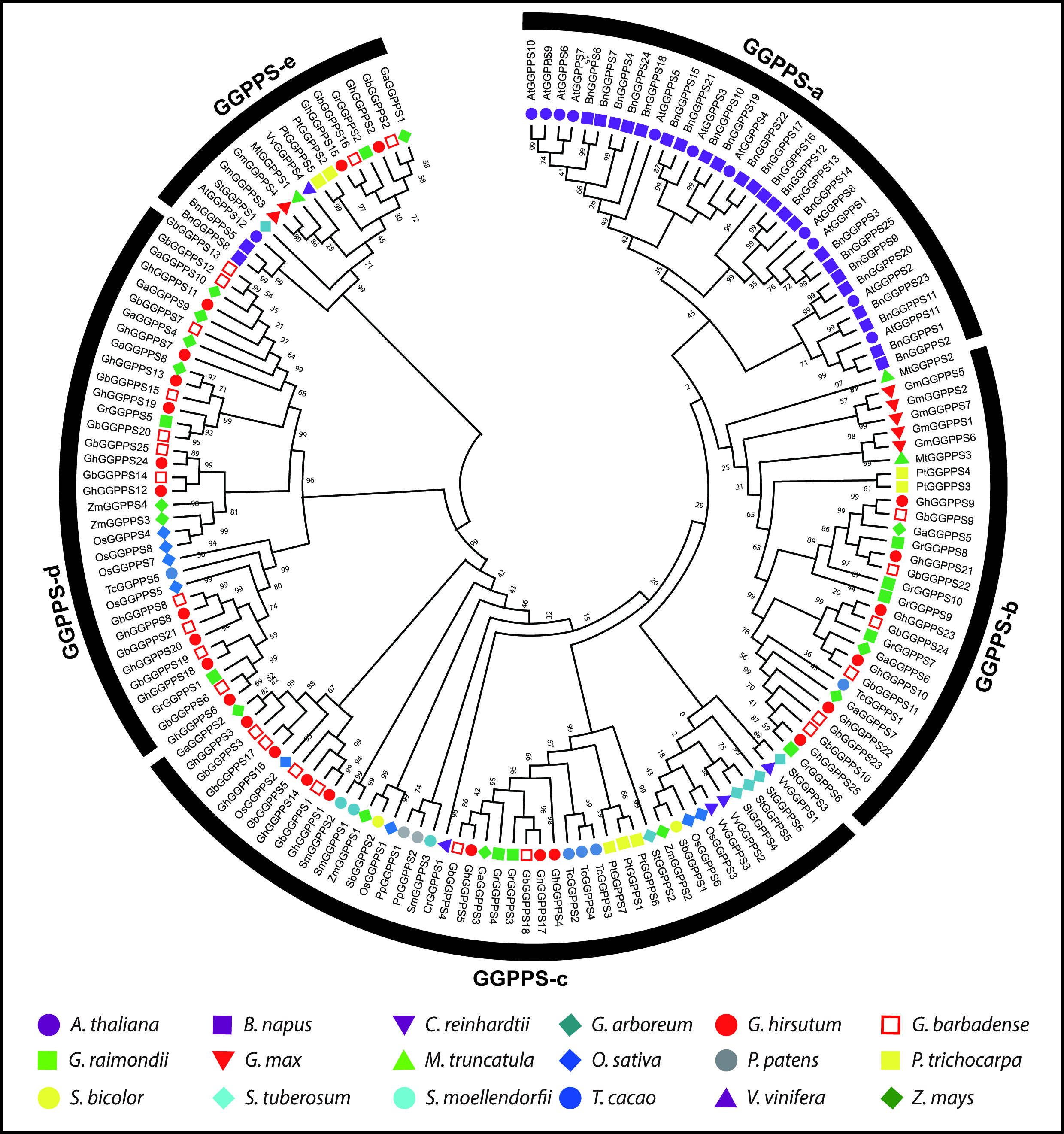

Supplement: Supplementary file 6 — Additional file 6: Figure S1. Evolutionary relationship among 159 GGPPS genes from 18 plant species using maximum evolution method. [file 12864_2020_6970_MOESM6_ESM.tif]

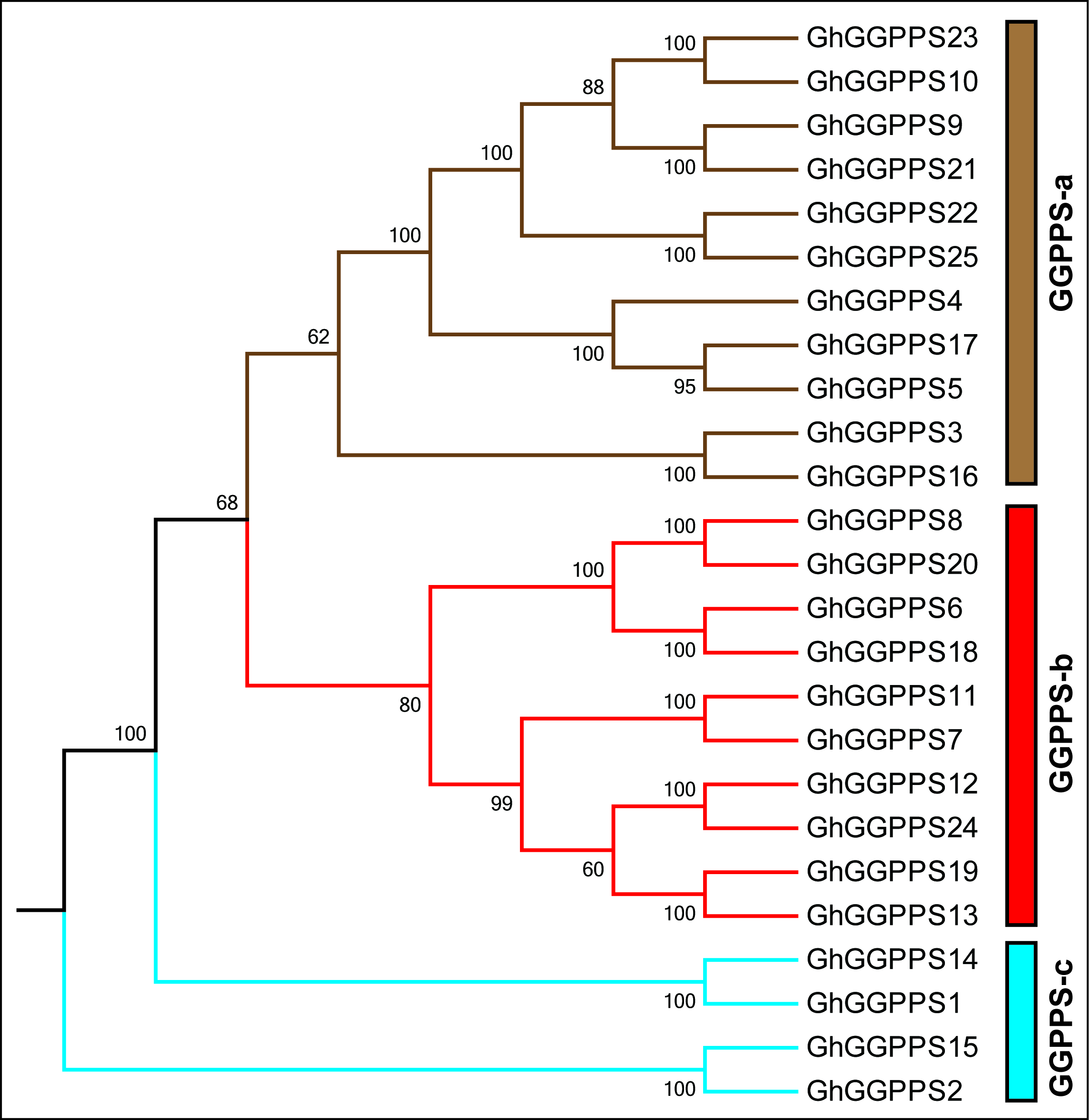

Supplement: Supplementary file 7 — Additional file 7: Figure S2. Evolutionary relationship of 25 GGPPS genes in G. hirsutum. Phylogenetic tree was constructed using MEGA software. [file 12864_2020_6970_MOESM7_ESM.tif]

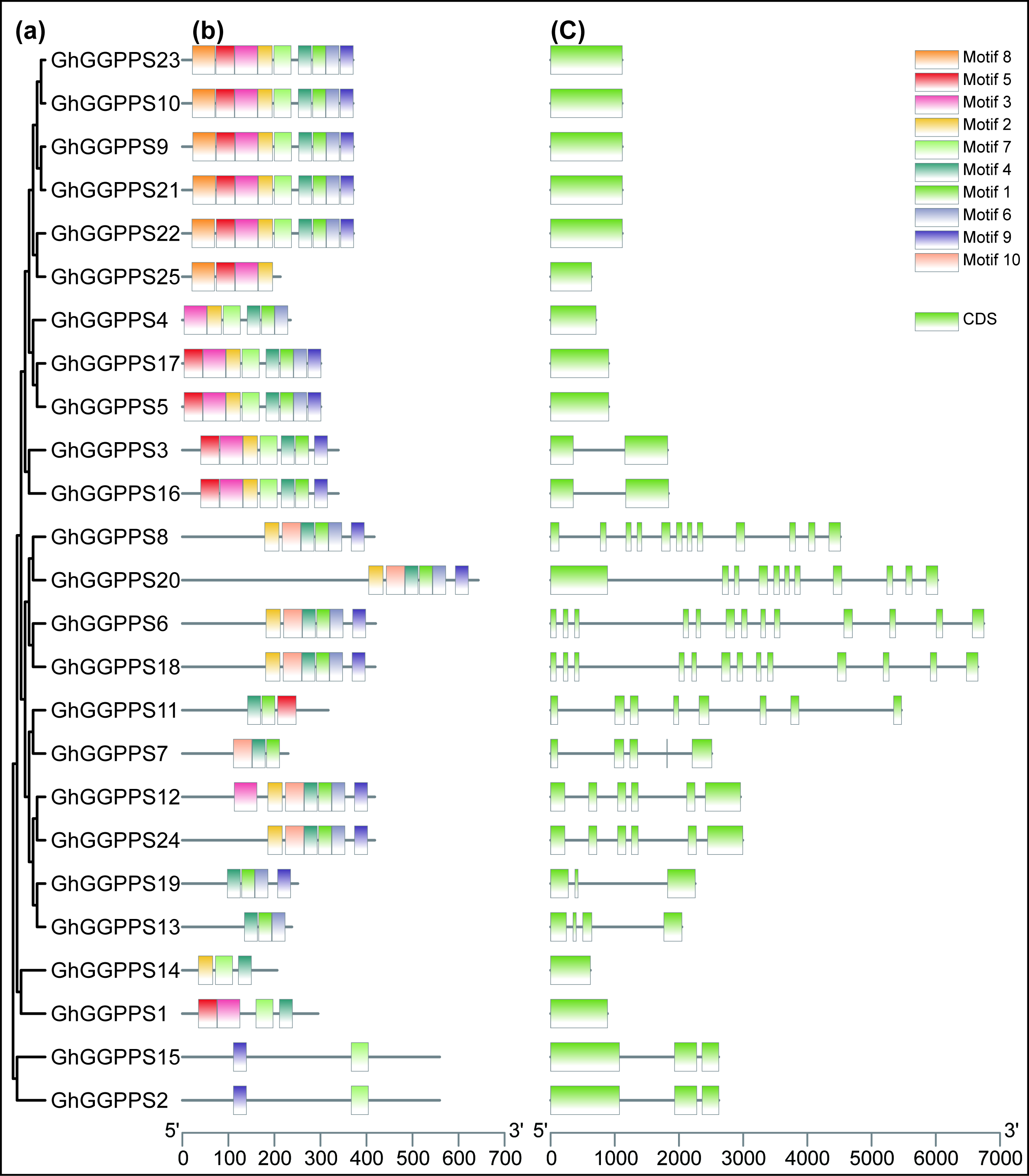

Supplement: Supplementary file 8 — Additional file 8: Figure S3. The gene structure analysis of GGPPS gene family in G. hirsutum. (A) The unrooted neighbor-joining (NJ) tree was constructed based on the GhGGPPS domains. (B) GGPPS gene family conserved protein motifs distribution. To identify different protein motifs of GhGGPPS gene family numbers (1–10) and different colors were given. (C) GhGGPPS gene family exon–intron structure was obtained by using GSDS 2.0. [file 12864_2020_6970_MOESM8_ESM.tif]

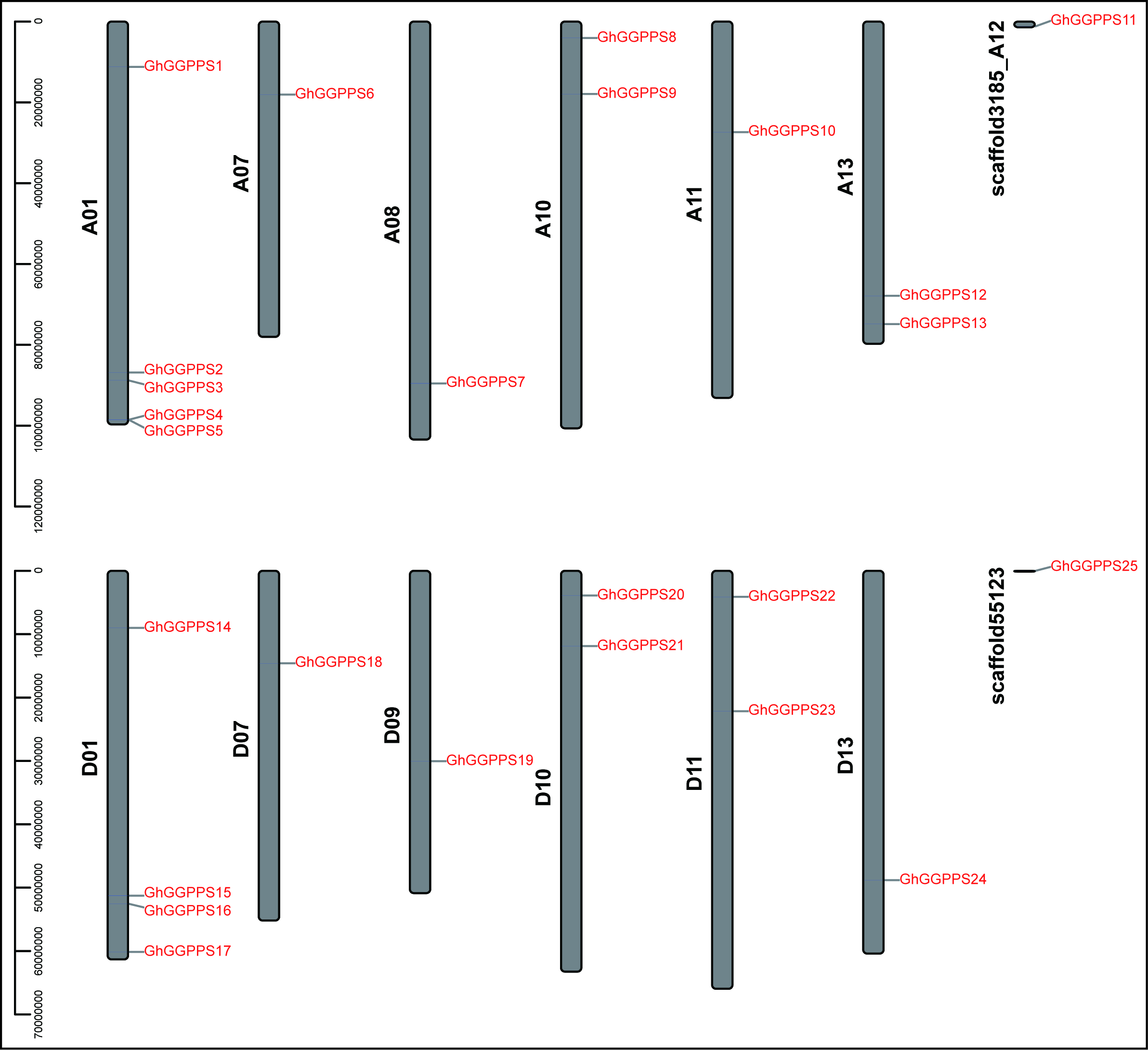

Supplement: Supplementary file 9 — Additional file 9: Figure S4. The distribution of GhGGPPS genes on the chromosomes of At and Dt sub genome of G. hirsutum. [file 12864_2020_6970_MOESM9_ESM.tif]

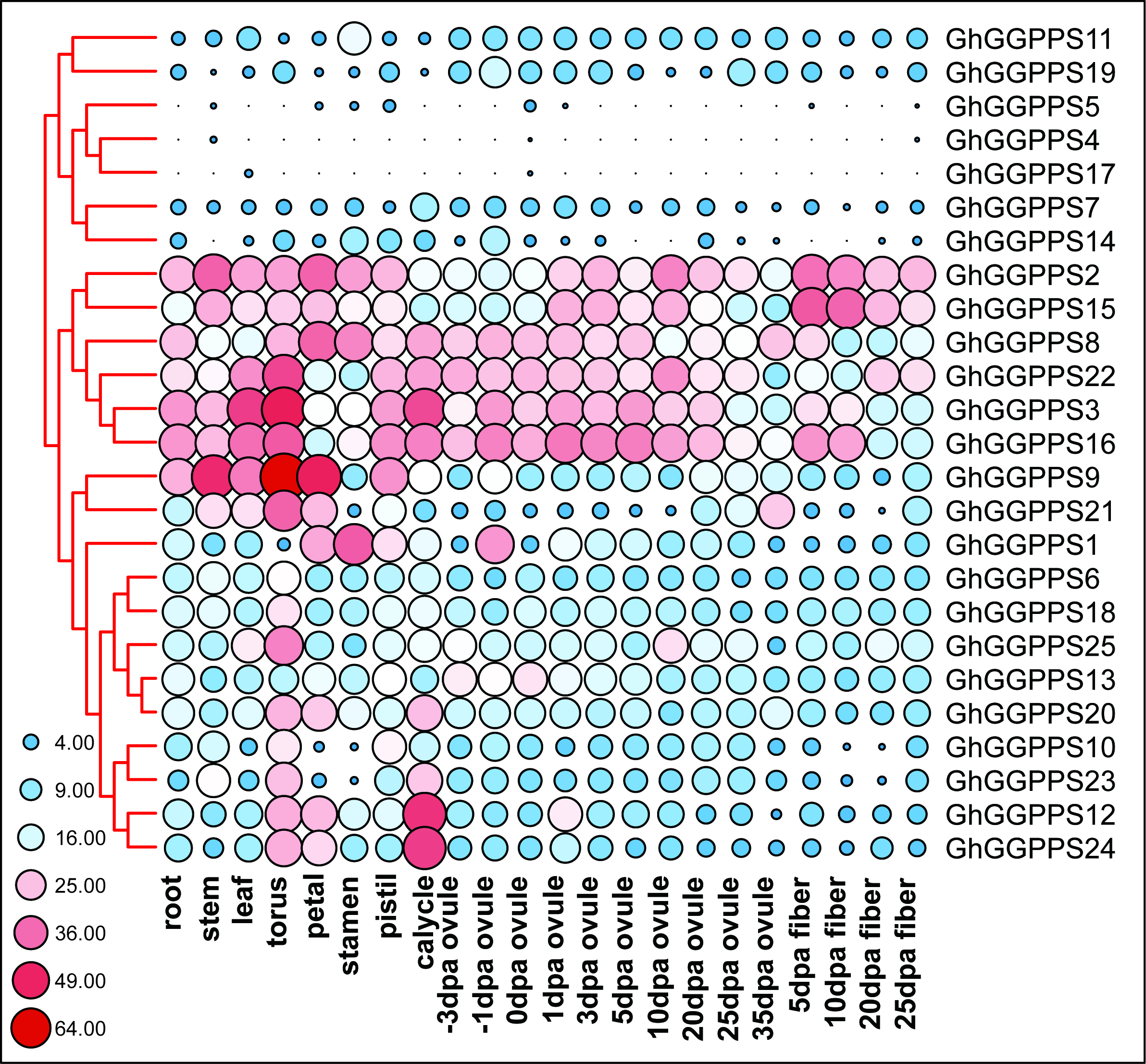

Supplement: Supplementary file 10 — Additional file 10: Figure S5. Heat map of GhGGPPS genes under different abiotic stresses. The RNA-Seq expression profiles of G. hirsutum were used for relative expression levels of GhGGPPS genes, gene expression level depicted in different colors on the scale. [file 12864_2020_6970_MOESM10_ESM.tif]

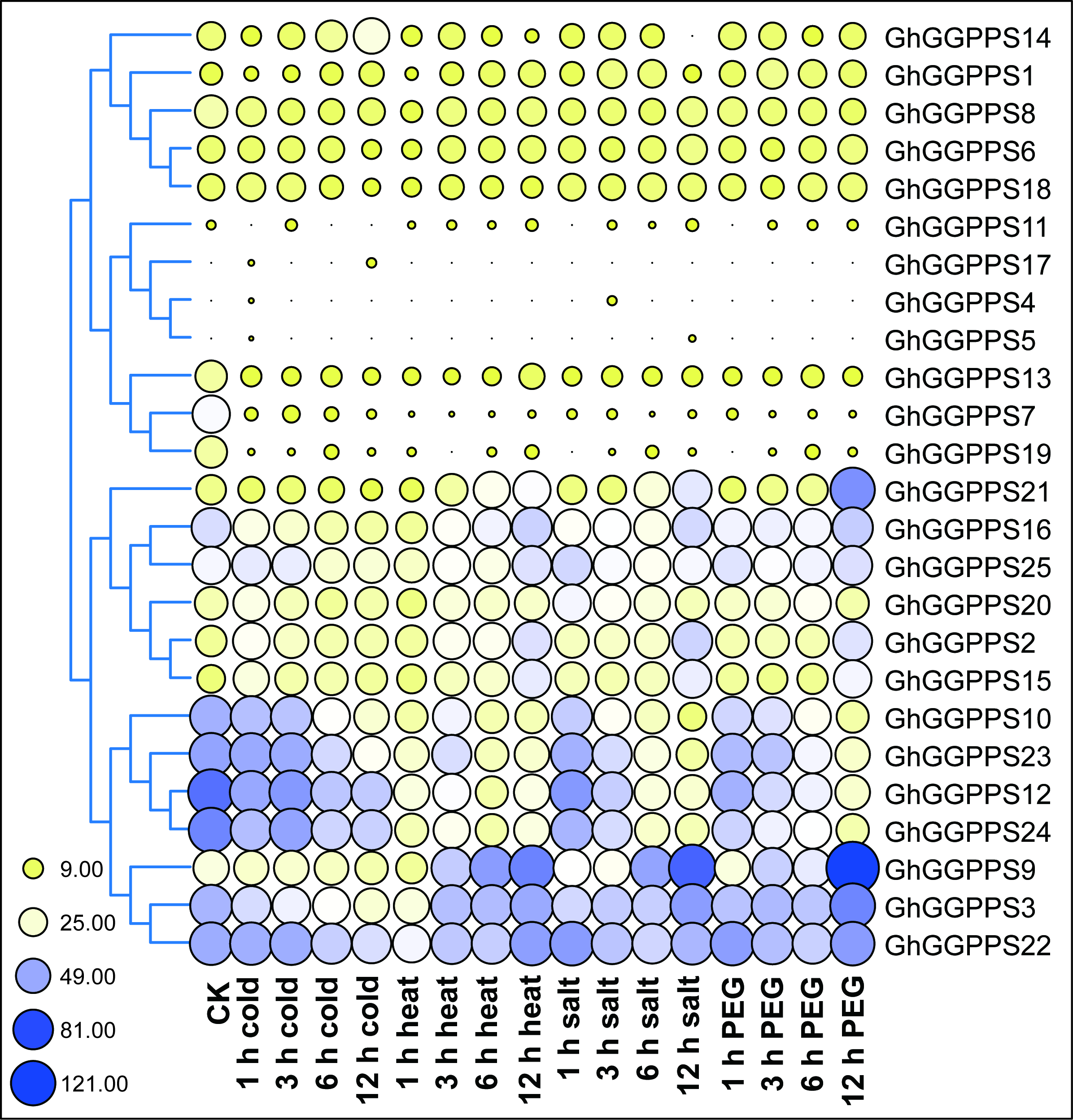

Supplement: Supplementary file 11 — Additional file 11: Figure S6. Expression levels of GhGGPPS genes in 22 tissues of G. hirsutum. RNA-Seq expression profiles were used to generate the heat map through Genesis software. [file 12864_2020_6970_MOESM11_ESM.tif]
